# The Discovery and Discoverer of Etherization

**Published:** 1863-07

**Authors:** 


### Art. VIII.—THE DISCOVERY AND DISCOVERER OF ETHERIZATION.

For the third time a Committee of the United States Congress has affirmed that Dr. Wm. T. G. Morton was the first and original discoverer of the vapour of ether being "a safe and practical anæsthetic agent." A renewed application of Dr. Morton for compensation, hitherto withheld, for the discovery and gift to his country and mankind of etherization, was, in the course of the third session of the present Congress, submitted to the Committee on Military Affairs and the Militia. In the report of this Committee on the subject recently published, Dr. Morton's claims are subjected to a full and exhaustive examination; and again it is conclusively established that to this gentleman alone belongs the honour of having first discovered the pain-subduing properties of ether vapour. The report adds no new facts to the history of the discovery, but it perhaps places in a clearer light than heretofore the utter worthlessness and unjustifiableness of the claims of the two chief contestants—Dr. Horace Wells and Dr. Charles T. Jackson. The accounts of the discovery current in this country, accord to these gentlemen greater credit in the matter than is due to them: it will be useful, therefore, to glance at some portions of the evidence laid before the committee.

The discovery of etherization dates from the 30th of September, 1846. On that day Dr. Morton induced a man named Eben Frost, suffering from violent toothache, to inhale the vapour of ether. The patient became unconscious almost immediately, and while in that state a firmly-rooted bicuspid tooth was extracted. Consciousness quickly returned, but the patient knew nothing of what had been done to him. "This," writes Dr. Morton, in a Memoir presented to the French Academy of Arts and Sciences, "I consider to be the first demonstration of this new fact in science." A like happy result followed the inhalation of ether in numerous other cases of tooth extraction (Dr. Morton, it will be remembered, practising as a dentist); and on the 16th of October, 1846, the anæsthetic was administered to a surgical case under the care of Dr. John C. Warren, in the Massachusetts General Hospital. While the patient was under its influence, a small vascular tumour was removed from the left side of the neck. The patient manifested some signs of sensibility during the operation, and although, when he had recovered his faculties, he averred that he had experienced no pain, the results were not considered altogether satisfactory. On the following day, however, a tumour of considerable magnitude was removed in the same hospital by Dr. Hayward, the patient being fully under the influence of the anæsthetic. This operation, as Dr. Bigelow says, "first showed conclusively the power of the new agent in averting the terrors of the surgical art."

Up to this period the nature of the anæsthetic had not been made known, and Dr. Morton had masked the ethereal fumes by adding to them certain aromatic odours. No further use was in consequence made of the anæsthetic in the Massachusetts General Hospital until the beginning of November, the surgeons declining to adopt a preparation of the precise nature of which they were ignorant. On the 6th of November, Dr. Morton, anxious to secure a final test, as it were, of the value of his anæsthetic, called upon Dr. Hayward, and begged him to make use of it in the case of a patient, one of whose limbs he was to amputate on the following day. Dr. Hayward informed Dr. Morton of the objections entertained by himself and fellow surgeons to the use of the anæsthetic. To this Dr. Morton frankly responded by communicating its nature, and on the 7th of November Dr. Hayward amputated the leg of a delicate girl of about twenty years of age, who had for a long time suffered from scrofulous disease of the knee-joint, Dr. Morton having first caused her to inhale ether until complete insensibility had been induced. The success of the anæsthetic was triumphant. When the patient recovered her consciousness, she was wholly ignorant that the operation had been performed, and for some time refused to believe the fact.

Prior to this crowning success, Dr. Morton, fully convinced of the anæsthetic properties of inhaled ether vapours, wrote to Dr. Wells, telling him that he had discovered a "preparation," by the inhalation of which a person could be thrown into a sound sleep. "The time required to produce sleep is only a few moments," he said; "and the time in which persons remain asleep can be regulated at pleasure. While in this state the severest surgical or dental operations may be performed, the patient not experiencing the slightest pain. I have perfected it, and am now about sending agents to dispose of the right to use it. I will dispose of a right to an individual to use it in his own practice alone, or for a town, county, or state. My object in writing to you is to know if you would not like to visit New York, and the other cities, and dispose of rights upon shares. I have used the compound in more than one hundred and sixty cases in extracting teeth, and I have been invited to administer to patients in the Massachusetts General Hospital, and have succeeded in every case." This letter was dated the 19th October, 1846, and on the next day Dr. Wells replied:—"Your letter dated yesterday is just received, and I hasten to answer it, for I fear you will adopt a method in disposing of your rights which will defeat your object. Before you make any arrangements whatever I wish to see you. I think I will be in Boston the first of next week—probably Monday night. If the operation of administering the gas is not attended with too much trouble, and will produce the effect you state, it will undoubtedly be a fortune to you, provided it is rightly managed."

It seems to be evident from this letter, the Committee observes, that at the time when it was written Dr. Morton did not "believe himself to be the discoverer of *an available anæsthetic agent.*"

Dr. Wells first appeared as a claimant, before the public, of being the earliest discoverer of a practically available anæsthetic, on the 7th December, 1846. He then advanced his claim in the *Hartford* (Connecticut) *Courant*, two years after the time of the pretended discovery, and limited his pretensions to having had a tooth drawn himself, and afterwards having performed a similar operation for "twelve or fifteen others" under the influence of nitrous oxide. No allusion was made by him to any experiment with ether, or to any use of nitrous oxide subsequently to the year 1845. After publishing this letter, he started for Europe on a speculation in pictures. Arriving in Paris he found the scientific world there all agog with the discovery of etherization But he at first advanced no claim with regard to it. In the meantime two enthusiastic friends in America had undertaken the defence of Dr. Wells's claim made in the *Hartford Courant*, but they superadded to his statements the important assertion that he had also experimented upon and showed the anæsthetic property of ether vapour. On this assertion being shown to Dr. Wells in Paris, he forthwith wrote to *Galignani*, and asserted that in his experiments he had made use of ether as well as of nitrous-oxide, and claimed the discovery of the anæsthetic power of both agents.

The curious process of growth observed in Dr. Wells's claim is paralleled by that observed in the evidence of the witnesses he adduces in support of it. No trustworthy testimony was produced by him of the use of ether; and how entirely his experiments with nitrous-oxide had disappointed the hopes with which he had prosecuted his researches on its anæsthetic properties, may be gathered (1) from the fact, that it is doubtful whether he had made use of the agent after an unsuccessful trial, in Boston, in the winter of 1844-5; and (2) from his having terminated an engagement of partnership with a Dr. Cooley, entered into on the faith of its success.

"The first intimation I had that Dr. Wells," says Dr. Cooley, "did not intend to carry out our partnership arrangement with me was when he informed me—several weeks after this arrangement was entered into between us—that he had just returned from Boston, where he had made a public experiment, which had proved a failure. He then said to me that he was disappointed in the effects of the gas, and that it would not operate as he had hoped and thought it would, as there was no certainty to be placed upon it, and consequently, he should abandon it, as he had so much other business to attend to, and as the gas would not operate in all cases alike, and therefore could not be trusted. He advised me to go on with my exhibitions, and thought I could make money out of them, and that, although he got through with his experiments in the business, he would assist me in any way he could in order that I might succeed in my lectures, and suggested to me to connect with my lectures and administering the gas mesmerism, and the use of a card of questions which he had prepared, so arranged that a correct answer could be given by a person in an adjoining room as to the time of day, &c., by the particular manner in which the question was asked. Feeling some confidence that by following his suggestions I should realize sufficient from the lectures to reimburse me for my time and expenses while in company with him, the matter was then dropped between us."

The Committee justly remark upon "the departure from the line of straightforward, undisguised truth" which characterizes Dr. Wells's letters with respect to, and his account of, his pretended discovery.

Dr. C. T. Jackson, in a memorial to Congress, dated the 29th of January, 1849, disputed Dr. Morton's assumption of the discovery of the anæsthetic properties of ether vapour, and asserted that the discovery was made by himself:—

"Without the knowledge of the said Morton, and without the co-operation or assistance of any person whomsoever, it was communicated by the undersigned (Dr. Jackson) to various persons, from the spring of 1842 to the 30th day of September, 1846, inclusive, and on the said 30th day of September was also communicated by the undersigned to the said Morton, he, the said Morton, being, previous to the said communication of the discovery to him, wholly ignorant of the anæsthetic properties and effects of sulphuric ether aforesaid; and whereas the undersigned did, also, on the 30th day of September, 1846, devise and commit to the said Morton the performance of an experiment for the verification of the said discovery, so far as the extracting of teeth is concerned; and whereas the said Morton, acting in strict conformity with the instructions, and upon the exclusive and expressly-assumed responsibility of the undersigned, did, to the extent of a painless extraction of a tooth, successfully verify the said discovery; and whereas the undersigned did, shortly afterwards, cause the discovery to be further verified by the surgeons of the Massachusetts General Hospital, in the first painless capital operation ever performed under the influence of the ether vapour; and whereas the signature of the undersigned to certain letters patent, taken out in the joint names of the undersigned and of the said Morton, declaring the discovery to be their joint invention, was obtained through the representation of Robert H. Eddy, Esq., of Boston, the solicitor by whom the said letters patent were procured, and copartner with the said Morton in the profits thereof, that the undersigned 'might lose all his credit as a discoverer,' if he did not consent to become a party to the said letters patent," &c.

The evidence is conclusive that long before the 30th of September, 1846, Dr. Morton was acquainted with sulphuric ether. Without dwelling upon the knowledge of the preparation which he must have picked up in the course of his attendance upon lectures while a student in the Massachusetts Medical College, and from reading; it is sufficient to state that Mr. Metcalf, a chemist, of "responsible scientific attainments," testifies to a conversation with Dr. Morton on the properties of sulphuric ether of so marked a character prior to July, 1846, that when he heard of the discovery of etherization, he being at the time in Europe, "he fixed it in his own mind that Dr. Morton was the man (discoverer) from this conversation." Dr. William P. Levitt, a pupil of Dr. Morton's, testified to having purchased sulphuric ether for Dr. Morton early in July, 1846. The Committee concludes that, "prior and subsequent to the 30th June, 1846, Dr. Morton was intent upon the discovery of some anæsthetic agent which would enable him to extract teeth without pain, and that he had faith and confidence that he was on the point of making the discovery." It is certain, however, that during his researches, and to hide their nature and tendency, he assumed complete ignorance of sulphuric ether.

The averment of Dr. Jackson that Dr. Morton's earlier experiments were performed under his instructions and upon his responsibility, is simply an impudent fabrication, without a shadow of confirmation. Indeed, he was not present at either of the experiments to which he refers, or at any experiment until the success of the anæsthetic agent had been fully assured. The first appearance of Dr. Jackson at an operation under the influence of ether vapour, at the Massachusetts General Hospital, was on the 2nd of January, 1847. No evidence is adduced by Dr. Jackson to show that at any time he possessed a greater knowledge of the properties of ether vapour than was to be found in ordinary medical text-books; and the Massachusetts Hospital Committee, in their report on the question of discovery, expressly said, that "down to September 30th, 1846, Dr. Jackson had discovered nothing that had not been known in print in London for some years." Dr. Jackson's first announcement of his pretended discovery was made in a sealed packet, dated the 13th of November, 1846, and transmitted to the French Academy of Sciences. He authorized the opening of this packet in a letter dated 1st of December, 1846; and when the sealed note was read, M. Velpeau observed that "the secret contained in the note which had been read was no longer a secret. The medical journals published in America and England had divulged it in the months of November and December. A letter from Dr. Warren, of Boston, communicated the information to him more than one month ago; and Dr. Willis Fisher, of the same city, proposed that he should try its effects at La Charité towards the middle of last December." It was not until November, 1851, in a letter to Humboldt, that Dr. Jackson vouchsafed a detailed account of his asserted discovery, first made in 1841-2!

Mr. Eddy's account of Dr. Jackson's association with Dr. Morton in the patent shows that when Dr. Jackson was informed that Dr. Morton was seeking to patent his discovery, Dr. Jackson saw Mr. Eddy and said, "he had some connexion with Dr. Morton" in making it. At first, it was arranged between Dr. Morton and Dr. Jackson that the latter should have five hundred dollars "for the information he had given Dr. Morton, if ten per cent. on the proceeds of the patent would produce the amount." But Mr. Eddy pointed out that unless Dr. Jackson's name was associated in the patent as joint discoverer, the patent might be impeached on account of the information he had given. Dr. Jackson hesitated for some time to associate himself with Dr. Morton in a patent, but at length assented. At the outset of the discovery, Dr. Jackson simply claimed credit and compensation for such advice and information as he had given Dr. Morton; he did not assert any rights of discovery.

For the rest we quote the summary of the evidence, and the conclusions of the Committee:—

"So long as the discovery was under test, and its result was uncertain, Dr. Jackson is unseen and unheard. When it became evident, from the two experiments at the hospital, that the discovery was of value, at the close of October, Dr. Jackson first appears, and then only for the purpose of claiming compensation of Dr. Morton for professional advice. He accepts five hundred dollars. His friend obtains for him ten per cent. of the net profits of the American patent. He next refuses to sign the European papers without receiving ten per cent. on the foreign patents. From this he rises to twenty per cent., and on the 28th of January he claims 'twenty-five per cent. both at home and abroad, as the least that in justice' can be offered him; and his counsel, of course with his sanction, speaks of the patent as one which, 'if sustained, promises to give to all parties large sums of money for their united co-operation.' He opens negotiations with Dr. Morton, through Mr. Hayes, for obtaining a joint patent in France, by the instrumentality of M. de Beaumont, whose letters to Dr. Jackson on this point were shown to Dr. Morton. After all hope of pecuniary benefit from the patent is at an end, he cancels the bond, and with a strange forgetfulness of all his previous conduct, comes out in the character of one who disdains pecuniary compensation. Not only so, but he seems determined that Dr. Morton shall receive no compensation. On the 20th November, 1847, the physicians and surgeons of the hospital (with one exception) prepared a memorial to Congress, setting forth the importance of this discovery, and praying the government to make a payment 'to those persons who shall be found, on investigation, to merit compensation,' on condition that the patent be given up. Knowing that this would result in an official inquiry into the discovery, Dr. Morton promoted it to the utmost of his power. Dr. Jackson, on the other hand, remonstrated against it, on the professed ground that he would submit his claims to no tribunal, and that, as the sole discoverer, he wished no reward beyond the gratitude of mankind.

"It is well known that an effort was made in London, by subscription, for a donation to the discoverer of the effects of ether. By letters to gentlemen in this country from friends in London, we are informed that a sum, estimated at 10,000*l.*, was considered as secured; but the controversy and doubt created by Dr. Jackson's communications to the French Academy caused it to be abandoned.

"Dr. Jackson speaks of Dr. Morton in terms of great bitterness. He assails his private character, declaring that it is infamous, and that in knowledge and intellect he is an ignoramus and an imbecile, not only not possessed of science, but mentally incapable of acquiring it; and that, while administering his anæsthetic vapour to the patients at the hospital, he was offensive to the faculty by reason of ignorance and quackery. Much of his letter to Baron Von Humboldt, which he filed before the committee of 1852 as his answer, for this reason would not be suffered to remain on the files of a court of chancery, but would be stricken out for scandal and impertinence. Your committee utterly refused, as stated above, to receive evidence of general character, or of particular accusation or defence for or against either of the parties not relevant to the issue; but as the charges advanced by Dr. Jackson against Dr. Morton in the letter above must remain on the files of the House and be printed with the proceedings of the committee, they deem it but just to say that these charges are not only not supported by, but are utterly inconsistent with, the current proofs in this case.

"The evidence presented with Dr. Wells's claim shows that dental operations were in several instances performed without pain by Dr. Wells under the influence of nitrous oxide, which had been before known in some cases to produce a total or partial asphyxia. It appears also that the vapour of sulphuric ether was thought of, discussed, and finally rejected by him; while the total abandonment of the use of nitrous oxide, and indeed of every other agent, shows that Dr. Wells's experiments were on the whole unsuccessful. He engaged in the search, and failed to find the object of his pursuit. He attempted and endeavoured assiduously to carry out the idea to practical results, but was not successful. There was great merit in the effort, but it proved a failure.

"Dr. Wells, therefore, in the opinion of your committee is not entitled to the honour of the discovery. He stopped half-way in the pursuit. He had the great idea of producing insensibility to pain, but he did not verify it by successful experiments. He mistook the means, and he unfortunately rejected the true anæsthetic agent as dangerous to life, and therefore did not make the discovery and give it to mankind. He did what Dr. Beddoes, Sir Humphry Davy, and Dr. Townsend had done about the close of the last century, but nothing more.

"But he had the signal merit of reviving the investigation, and, probably, of hastening the discovery. If an idea connected with the subject lay dormant in the mind of any one, his attempt was well calculated to awaken it into life. When, in the fall of 1844, he made his public attempt, in Boston, to produce anæsthesia during a dental operation, by the use of nitrous oxide, if Dr. Jackson had indeed made and perfected this discovery, and felt an abiding confidence in its truth, who can doubt that he would have availed himself of that occasion, or have been reminded by it, to make for himself another, at an early day, of publicly exhibiting and testing the true anæsthetic agent?

"The question of discovery, which your committee has thus endeavoured to examine, was every way proper to be tried and settled by intelligent men, as a jury of the vicinage, which was proposed by Dr. Morton and refused by Dr. Jackson. But it was finally tried by a most appropriate tribunal, the trustees of the Massachusetts General Hospital, at which the first public exhibition of this pain-destroying power was made, and where its effects were first witnessed by an admiring audience. The question of discovery was tried before these men—trustees of a scientific corporation, to whom Dr. Jackson was well known as a distinguished member of the medical faculty, and to whom Dr. Morton, prior to the discovery, and the contest to which it led, was known only as a young man of energy and enterprise. And this board, composed of men whose names would do honour to any scientific institution, presently after the discovery, near the time and at the place where it occurred, gave, by a unanimous voice, its honour to Dr. Morton. One year after they reviewed their decision, at the request of Dr. Jackson, and unanimously confirmed it.

. . . . "Before this tribunal neither time, place, nor circumstance permitted bold and confident assertion to be mistaken for truth. With this award we think Dr. Jackson, Dr. Wells, and the scientific world should have been satisfied. It is, in the opinion of your committee, entitled to great weight. It was the *first*, and ought to have been the *only* contest. Our enlightened system of jurisprudence forbids, except under extraordinary circumstances, *a second trial of questions of fact.* It forbids it as a guard against the danger incident to repeated investigations, that truth will be overborne by artfully manufactured evidence.

"Therefore, even if the evidence before your committee rendered the question of fact doubtful, which it does not, they would hesitate long before they would overrule the decision of the trustees of the Massachusetts General Hospital.

"It is also a subject of much gratification to this committee to be able to concur in the opinion of the former committees of the House, from whose very able reports they have extracted so largely. They did not, however, feel themselves bound by either the one or the other, but gave the subject for themselves a full and careful consideration. But they are the more satisfied with the conclusions to which they have come because of their concurrence with such high and unexceptionable authorities.

"Dr. Jackson appeals to the Academy of Arts and Sciences at Paris, and claims that the learned body has decided the question of discovery in his favour by awarding him the 'Monthyon prize for the greatest medical discovery,' and that their decision ought to be taken as final and conclusive. . . . .

"But the Academy of Arts and Sciences at Paris did not, as it appears, award to Dr. Jackson the honour of the discovery, either directly or indirectly, by awarding him '*the Monthyon prize for the greatest medical discovery.*' Your committee have inspected the official awardments exhibited by the parties, and find that the award to Dr. Jackson was '*one of the prizes* of medicine and surgery of the Monthyon foundation.'

"Upon a full examination of the whole case so far as time and means were afforded to your committee, they have come to the conclusion:—

"1st. That Dr. Horace Wells did not make any discovery of the anæsthetic properties of the vapour of sulphuric ether, which he himself considered reliable, and which he thought proper to give to the world. That his experiments were confined to nitrous oxide, but did not show it to be an efficient and reliable anæsthetic agent, proper to be used in surgical operations and in obstetrical cases.

"For the rest your committee have come to the same conclusions that were arrived at by the trustees of the Massachusetts General Hospital at their meeting in January, 1848, and re-considered and confirmed in 1849, and adopted by the former committee of the House—viz.,

"2nd. That Doctor Jackson does not appear at any time to have made any discovery in regard to ether which was not in print in Great Britain some years before.

"3rd. That Doctor Morton, in 1846, discovered the facts before unknown, that ether would prevent the pain of surgical operations, and that it might be given in sufficient quantity to effect this purpose without danger to life. He first established these facts by numerous operations on teeth, and afterwards induced the surgeons of the hospital to demonstrate its general applicability and importance in capital operations.

"4th. That Doctor Jackson appears to have had the belief that a power in ether to prevent pain in dental operations would be discovered. He advised various persons to attempt the discovery, but neither they nor he took any measures to that end, and the world remained in entire ignorance of both the power and safety of ether until Doctor Morton made his experiments.

"5th. That the whole agency of Doctor Jackson in the matter appears to consist only in his having made certain suggestions, which aided Doctor Morton to make the discovery—a discovery which had for some time been the object of his labours and researches.

"Though it was but 'a single step, and that a short one,' from the daily walks of science to this great discovery, yet the scientific world admits that the step was never taken prior to the 30th of September, 1846; and the discovery, when in fact made, was instantly appreciated, and hailed by the surgical profession with the most exalted enthusiasm, almost with shouts of rapture. The committee have thought proper to annex the following extracts from the records of the Patent Office:—

"'I have therefore, in consideration of one dollar, to me in hand paid, the receipt whereof I do hereby acknowledge, assigned, set over, and conveyed, and by these presents do assign, set over, and convey to the said Morton and his legal representatives, all the right, title, and interest whatever which I possessed in the said invention or discovery, a specification of which I have this day signed and executed in conjunction with him, for the purpose of enabling him to procure a patent thereon.

"'And I do hereby request the Commissioner of Patents to issue the patent to the said Morton *in his name*, and as my assignee or legal representative, to the extent of all my right, title, and interest whatever in the said invention or discovery.

"'In testimony whereof, I have hereto set my signature and affixed my seal this twenty-seventh day of October, one thousand eight hundred and forty-six.

"'CHARLES T. JACKSON.

"'Witness: R. H. EDDY.'"

If, then, to Dr. Morton belongs the honour of the greatest discovery which has been made since the discovery of vaccination, he has unhappily the bare honour alone. In pursuing and making known his researches, he lost his source of livelihood and exhausted his means. But for the generous sympathy of his professional brethren in America, he would have been reduced to complete poverty. He benefited the world at the expense of his own ruin. His patent yielded him nothing; it was early set at defiance, not only in civil life, but also in the army and navy. The account of his losses which Dr. Morton laid before the Committee is curious. It is as follows:—

*William T. G. Morton in account with his discovery, from October,* 1846, *to* 1863, *Dr.*

| | |
|---|---|
| 1847 : Translating and publishing several editions of report of American cases and other documents for use abroad, with expenses of their transmission, with ether and apparatus, together with postage, freight duties, and cost of foreign correspondence . . . | $2,860 |
| Various professional and scientific men, for services rendered in promulgating discovery, collecting cases, demonstrating value where opposition existed, together with their travelling expenses* . | 3,162 |
| Various literary gentlemen, for procuring favourable opinions of the press, preparations of replies, and other papers, with salary of private secretary . . | 2,100 |
| Printing and publishing of papers and pamphlets during introduction of discovery, newspaper articles, circulars, &c., &c. . . . . . . . . . . . . | 4,326 |
| Ether distributed among professional men . . . . | 2,640 |
| Apparatus distributed among professional men . . | 3,060 |
| Apparatus remaining unsold . . . . . . . . . | 2,000 |
| Deficiency in patent account . . . . . . . . . | 2,000 |
| Hotel and travelling expenses from time of discovery to date, with hack-hire, telegraphing, expressing, &c., &c., $1,000 per annum . . . . . . . . . | 17,520 |
| Printing and publishing, &c., from 1847 to date . . | 5,999 |
| For services, to Messrs. Webster, Choate, Carlisle, Curtis, Whiting, Dana, Cornwall, and others, together with advice and other services rendered . | 12,550 |
| Expenses for testimony *in perpetuum* at Boston, Hartford, and other cities, including examination of witnesses, drawal of papers, travelling expenses, &c. | 4,870 |
| Recording of testimony in Boston . . . . . . . | 169 |
| Cost of manufacture of nitrous oxide gas for experiment at Washington† . . . . . . . . . . | 50 |
| Expense for testimony of medical and surgical, literary, scientific, and other gentlemen, also assistants to aid in collecting the same, stationery, copying, printing, binding, postage, and distributing the same to still further refute opponents, overcome opposition; expense of the suit suggested by the President of the United States as a pre-requisite to paying Dr. Morton . . . . . . . . . . . | 10,255 |

* These were usually young physicians, who, whenever a fatal case or non-success was reported, as was constantly being done during the days of opposition, were instructed in the method of administration, and sent by Doctor Morton to counteract the feeling. Visits were paid by them to New York, Philadelphia, Montreal, and even some of the cities of the far west.

† This was for the trial demanded by Dr. Morton to disprove to the satisfaction of the congressional committee the claim of Horace Wells, by showing the inert character of the agent for the purposes of anæsthesis.

| | |
|---|---|
| Interest . . . . . . . . . . . . . . . . . . | $42,000 |
| Sacrifice of income 17 years, proved to be worth $10,000 per annum, but put down in this account at only one-half that sum . . . . . . . . . | 85,000 |
| Total . . . . . . . . . . . . . . . . . | 200,561 |

On the 23rd of February, 1849, Dr. Morton filed a petition in the House of Representatives, asking compensation for the services he had rendered to his country in the discovery and surrender to the public use of etherization. The subject was fully investigated, and the Committee, after a careful consideration of its merits, expressed the opinion that the petitioner was the true discoverer, but referred the question of compensation to the House, and nothing more was done about it.

In 1852 Dr. Morton again petitioned, and a Select Committee again reported in his favour, and a bill was prepared for his relief, but the business of the House rendered it impossible to present it. The Chairman of the Committee feeling the great wrong done to Dr. Morton, then attempted to get the bill attached to the military and naval Appropriation Bill for the then ensuing year. The proposition was considered by the Naval and Military Committees of the Senate, and each, by resolution, recommended an amendment to the Army Appropriation Bill in the following words:—"To enable the President of the United States to procure the surrender of the patent issued to Wm. T. G. Morton on the 12th of November, 1846, for his discovery of the anæsthetic properties of sulphuric ether, one hundred thousand dollars." The amendment was lost, owing to its being out of the usual course of business. The subject was once more submitted to the Senate in 1852, and again in 1854. Each time it was recommended * that an appropriation should be made for the benefit of the discoverer, the title to discovery to be settled in a court of justice; and it was further proposed that this recommendation should be introduced as an amendment to the Army Appropriation Bill. The amendment both times passed the Senate, but was lost in the House. Dr. Morton then petitioned the President directly. The President at first seemed favourably impressed towards the petitioner, and promised to forbid the use of ether in the naval or military services, or pay a stated compensation; but afterwards, on the suggestion of Jefferson Davis, then Secretary of War, he declined to act until the judgment of a judicial tribunal should determine the question of right. Dr. Morton, therefore, on the President's suggestion, brought a

* This recommendation was made at the suggestion of Dr. Morton, it being found that there was not sufficient time to discuss the questions at issue fully in the two Houses.

suit against a United States officer for breach of patent right; and after two years and a large expenditure of money, obtained a judgment against him. But when this judgment was delivered a new administration had come into power, and the head of the Treasury department, when the record of judgment was produced, refused to carry out the President's desires. The Executive in fact refused compensation, and continued to use the discovery as before. In the meantime the patent was drawing to a close, and Dr. Morton determined to apply for an extension, hoping that the Executive would yet be induced to recognise and respect his rights; but this extension was denied, because of some technical formality with which he could not comply. "Your committee, however," says the Committee last sitting, "considers his claim as valid against the United States, in equity and good conscience, as it would have been if no such difficulty had been interposed, and the patent had been extended according to usual practice of the office in ordinary cases."

Now it was that the medical and surgical faculties stepped forward to aid Dr. Morton, amidst his anxieties, disappointments, and losses. The subscriptions they procured were most liberal, and the late Committee expresses the warmest admiration of the sympathy and support thus extended to the unfortunate discoverer in his disheartening progress; but the sum obtained fell far short of what was necessary to indemnify him for his expenditure of time and money in pursuit of the discovery, and "in giving it to the public under conditions which would command public confidence."

Once more, then, Dr. Morton has appealed to the Senate; once more a Committee of Congress has reported in his favour, and the report terminates as follows, referring in the first place to the assistance which Dr. Morton has received from the medical profession:—

"Nor is it just that this single profession should take upon itself, by its extraordinary efforts, the burden of rewarding and sustaining a discovery by which the nation has been, and is now especially, so largely benefited. Who shall estimate its value to the army and navy of the United States? For what sum would the government now consent for a single year, or even after a single battle, to forego its use? It is in proof before us that Dr. Morton himself administered his *nepenthe* to more than a hundred wounded soldiers fresh from the battle-field of Fredericksburg, and with three minutes to the man, and without a single failure, prepared them all for a painless operation with the probe and the knife. His *nepenthe* is used in all operations in the army and navy, and we are safe in saying that no sum which could be named would induce the United States to forego its use.

"It was used for fourteen years, as far back as the Mexican war, and down to the expiration of the patent. To his legal right to indemnity and compensation for *this*, no one who regards the mandates and prohibitions of the constitution can doubt. The legal liability of the United States for this has been judicially determined in the suit above referred to. This is independent of all consideration of merit in the discovery. It is a legal right; it is property; and it is all the property the petitioner has left to him. It has been taken by the United States, and it has been applied to public use. It is debt due to Dr. Morton, and long withheld. He is also, in the opinion of your committee, entitled equally to compensation for its use to the present time, and hereafter, down to the expiration of an extended patent, according to ordinary law and the usage of the department; and he is entitled, not in strict law or, perhaps, in legal equity, but in sound political morality, to liberal consideration for the priceless service which he has rendered to his country and its people in every condition of life. We are satisfied that Dr. Morton is the discoverer. We think him entitled to liberal compensation and reward, in accordance with the usages of this and other governments in such cases.

"The only question as to this claim is the amount. A bill twice passed the senate appropriating a hundred thousand dollars to the discoverer. Former committees and heads of departments, at a time when there was not a tithe of the evidence that the use of the discovery by the United States now furnishes of its value to the government, reported in favour of and recommended appropriating one hundred thousand dollars to enable the President of the United States to procure the surrender of Dr. Morton's patent.

"The medical faculty say 'its value is such that, if it were only to be purchased with large sums of money, millions of dollars would readily and properly be paid by persons who are subjects of the pain the discovery is competent to avert or relieve;' and 'that Dr. Morton ought to have a monument of gold as high as Trinity Church steeple.' Though it may be utterly impossible to determine the proper bounds within which merit is to be rewarded in a case like the present, in which an humble individual is the donor and the whole nation the recipient, we can appropriate a sum of money which will reimburse and indemnify him for expenses and sacrifices in bestowing this boon, and place his future life beyond the reach of poverty, and in this manner do justice to ourselves.

"An account stated, supported by satisfactory evidence, shows that Dr. Morton has expended in money, and time, and sacrifice of professional business, more than two hundred thousand dollars in discovering, defending the discovery and his rights thereto, and perfecting and giving the nation this pain-destroying agent.

"Your committee are of the opinion that some compensation is due, but they report these facts for the information of the senate, without any recommendation."

We trust that these words will fall on a less barren soil than those of previous committees. But if Congress again turns a deaf ear to Dr. Morton's petition, should England, nay Europe, stand idly gazing upon this great wrong?

---

